# Effects of clipping of flight feathers on resource use in Gallus gallus domesticus

**DOI:** 10.1098/rsos.211561

**Published:** 2022-03-02

**Authors:** Renée Garant, Bret W. Tobalske, Neila BenSassi, Nienke van Staaveren, Dan Tulpan, Tina Widowski, Donald R. Powers, Alexandra Harlander-Matauschek

**Affiliations:** ^1^ Department of Animal Biosciences, University of Guelph, 50 Stone Road E, Guelph, ON N1G 2W1, Canada; ^2^ Division of Biological Sciences, University of Montana, 32 Campus Drive, Missoula, MT 59812, USA; ^3^ Department of Biology, George Fox University, 414N Meridian St, Newberg, OR 97132, USA

**Keywords:** feather loss, flapping flight, locomotion, wing damage, wing wear, bird

## Abstract

Ground-dwelling species of birds, such as domestic chickens (*Gallus gallus domesticus*), experience difficulties sustaining flight due to high wing loading. This limited flight ability may be exacerbated by loss of flight feathers that is prevalent among egg-laying chickens. Despite this, chickens housed in aviary style systems need to use flight to access essential resources stacked in vertical tiers. To understand the impact of flight feather loss on chickens' ability to access elevated resources, we clipped primary and secondary flight feathers for two hen strains (brown-feathered and white-feathered, *n* = 120), and recorded the time hens spent at elevated resources (feeders, nest-boxes). Results showed that flight feather clipping significantly reduced the percentage of time that hens spent at elevated resources compared to ground resources. When clipping both primary and secondary flight feathers, all hens exhibited greater than or equal to 38% reduction in time spent at elevated resources. When clipping only primary flight feathers, brown-feathered hens saw a greater than 50% reduction in time spent at elevated nest-boxes. Additionally, brown-feathered hens scarcely used the elevated feeder regardless of treatment. Clipping of flight feathers altered the amount of time hens spent at elevated resources, highlighting that distribution and accessibility of resources is an important consideration in commercial housing.

## 1. Introduction

Flight feathers make up the majority of the area of bird wings and tail and are key to generating lift for weight support and thrust during flight [1]. This key role in flight makes the maintenance of well-shaped and intact flight feathers an integral component of flight performance and survivability in wild birds [2]. However, the flight feathers of wild birds are often worn, damaged or removed due to predator attacks, disease (e.g. parasites), poor weather, nutritional challenges, collisions and abrasions from the environment [3]. As feathers are dead structures and cannot repair themselves, birds will replace worn and missing feathers through an energetically demanding and lengthy moulting process [4–6].

The loss or damage of flight feathers reduces wing area, changes the wing's size and shape, impairs wing integrity, and increases wing loading [7]. Such significant changes to this important appendage can compromise a bird's flight pattern and trajectory, in addition to increasing the energetic cost to support body weight during flight [8]. Following wing damage or loss, birds may increase flapping frequency and wing beat amplitude to accommodate missing wing feathers [8,9]. For birds of prey, the loss of only one feather is enough to substantially reduce flight performance and hunting ability [2]. Birds that are forcibly grounded in such a manner are more vulnerable to predation and are less successful in finding food [10].

In contrast with many flying species of birds, ground-dwelling species, such as chickens, will only fly infrequently, mainly to avoid predation and locate safe sleeping or roosting spaces [11–13]. Domestic birds kept for egg-laying (*Gallus gallus domesticus*) navigate their living space by walking, running, climbing, wing-assisted running and short-burst flapping flight [14–16]. Despite being ground birds, laying hens are highly motivated to roost using an elevated perch [17]. As roosting in elevated spaces is a behavioural priority, perches of varying heights are commonplace in coops for backyard chickens or single-tier systems for commercial hens. By contrast, feed and nest-boxes are typically placed low to the ground. In multi-tier systems, the upper tiers contain essential resources, such as feeders, drinkers and nest-boxes [18]. This environment requires hens to access elevated spaces to feed, drink, perch and lay eggs. Laying hens must, therefore, be able to perform flapping flight to move up and down. A recent study by Léon *et al*. [19] shows that laying hens use the maximum amount of power possible during flapping flight trajectories. Consequently, this increases the risk of losing control and incurring injuries when descending from higher elevations.

Bird-to-bird pecking causes feather damage and loss. The prevalence of pecking behaviour has been reported at rates of 15–95% among laying hen flocks and can target wing and tail feathers [20]. Furthermore, abrasions or collisions within a housing environment may also lead to poor feather cover [21–24]. Direct consequences of wing feather damage in laying hens include the loss of aerodynamic surface, a decreased ability to stabilize on perches and impaired balance [25]. Given that laying hens are ground-dwelling birds that already operate their wings at the maximal power output [19], feather damage may cause them to become constrained to the ground and less capable of accessing elevated spaces. In turn, this would suggest that individuals suffering from feather cover loss may be less inclined to seek out the resources they need. This may be especially true as chickens age, and continuous feather pecking can lead to fully denuded hens with no feathers.

The present study investigates experimental wing feather clipping on feeder and nest-box choices of laying hens. We provided ground-level and elevated feeders and nest-boxes to laying hens (two strains) either with clipped or unclipped flight feathers. We monitored individual birds' use of these locations using a radio-frequency identification (RFID) system. We hypothesized that flight feather clipping would reduce the use of elevated resources. We anticipated this effect to be accentuated in heavier, brown-feathered birds when compared to their white-feathered counterparts. The data from this study will inform appropriate and safe spatial distribution of resources in coops for backyard chickens and commercial farms where feather loss and damage are prevalent, especially among white- and brown-feathered birds whose differing morphologies may require alternate resource placement, ultimately improving the health and welfare of chickens.

## 2. Material and methods

### 2.1. Animals and housing

One hundred and twenty female domestic chickens (*Gallus gallus domesticus*) composed of two strains aged 39 weeks (brown-feathered) and 34 weeks (white-feathered) were housed in 12 identical floor pens (183 L × 244 W × 290 H cm) with 10 birds of one strain per pen. Brown-feathered hens had an average body weight of 2090 ± 26 g and white-feathered hens 1765 ± 26 g. Prior to this experiment, birds were reared under commercial management conditions in enriched aviary housing until 23 weeks of age (brown-feathered) and enriched colony housing until 18 weeks of age (white-feathered).

**Figure 1.**
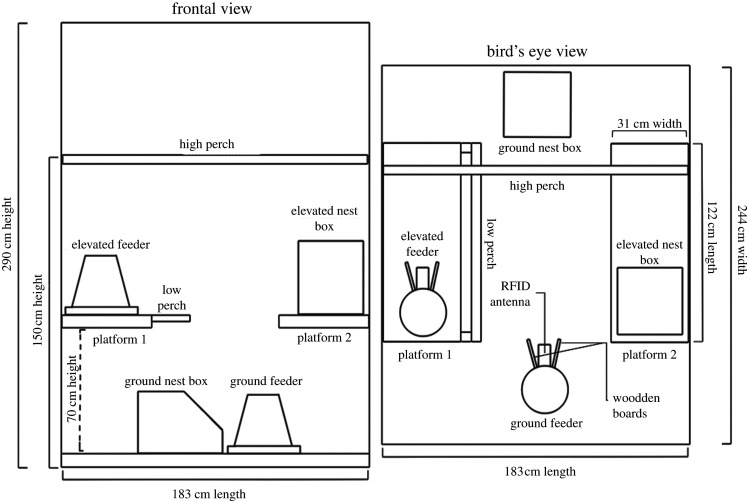
Diagram of experimental pen set-up including two views: frontal and bird's eye view. Each hen was housed in a floor pen (183 L × 244 W × 290 H cm) furnished with two elevated platforms (122 L × 31 W × 70 H cm), a high perch (244 L at 150 H cm), a low perch (122 L at 70 H cm), two 5 kg hanging feeders fixed with two wooden boards and an RFID antenna, and two individual nest-boxes each containing an RFID antenna.

Hens were on a 14 : 10 light : dark cycle in a ventilated, windowless room at the Research Station of the University of Guelph. Pens (figure 1) were littered with wood shavings and fixed with two elevated, slatted platforms (122 L × 31 W at 70 H cm, to mimic aviary height) symmetrically fixed to opposite pen walls. There was one low wood perch attached to the long side of platform 1 (122 L at 70 H cm) in addition to a high wood perch spanning the width of the pen (244 L at 150 H cm). Opaque boards were placed along adjoining pen walls to prevent social learning and physical contact between neighbouring hens/pens. Hens had *ad libitum* access to 10 nipple drinkers, two feeders and two nest-boxes (see ‘Behavioural activity measurements’ for more information on the feeders/nest-boxes). Hens were identified with numbered (1**–**120) ‘backpacks’ made of soft silicone rectangles (14.5 **×** 6 **×** 0.2 cm) and two elastic straps attached with eyelets [26].

### 2.2. Treatments applied to flight feathers

**Figure 2.**
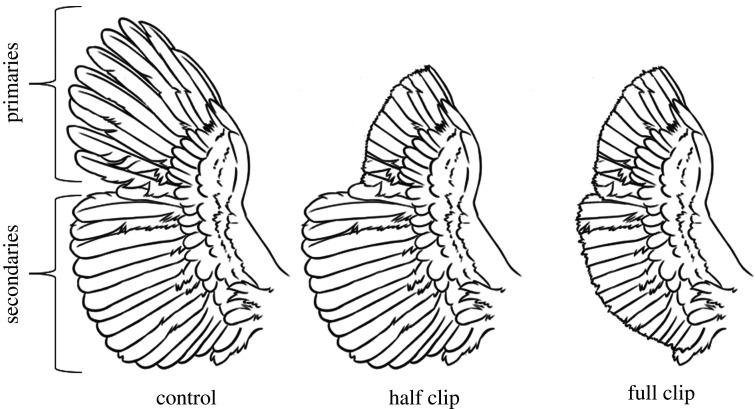
Diagram of treatments applied to flight feathers of laying hens (*n* = 120) at the end of week 0, following baseline measurement acquisition. Treatment groups included the control group in which all flight feathers were left intact (4 hens/pen), the half clip group in which the primary flight feathers were trimmed (3 hens/pen) and the full clip group in which all of the primary and secondary flight feathers were trimmed (3 hens/pen).

Hens were selected at random per pen to receive one of three treatments (figure 2) to both wings: flight feathers kept intact and wing loading was approximately 194.6 ± 10.7 kg m^−2^ (brown-feathered) and approximately 162.4 ± 11.5 kg m^−2^ (white-feathered) (Control, four hens/pen); primary flight feathers clipped, resulting in a 32.5% wing area loss and wing loading was approximately 252.7 ± 14.0 kg m^−2^ (brown-feathered) and approximately 249.1 ± 29.1 kg m^−2^ (white-feathered) (Half clip, three hens/pen); or primary and secondary flight feathers clipped, resulting in a 55.4% wing area loss and wing loading was approximately 354.1 ± 48.8 kg m^−2^ (brown-feathered) and approximately 378.9 ± 53.9 kg m^−2^ (white-feathered) (Full clip, three hens/pen). One person securely held the bird while a second person spread the wing feathers and clipped them using sterile scissors adapted from Harrison & Flinchum [27]. The coverts were used as a cutting guideline, and special attention was given to avoid newly forming blood feathers [28]. All birds were returned to their pens following treatment.

### 2.3. Behavioural activity measurements

**Figure 3.**
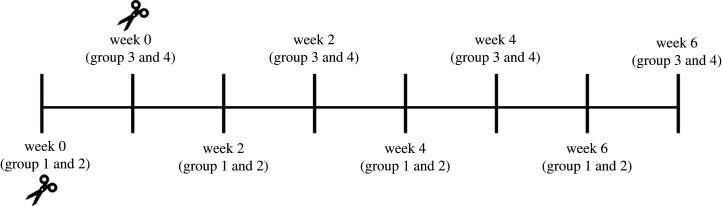
Experimental timeline. The experiment took place over the course of eight weeks. RFID equipment was rotated among 12 pens that were grouped into one of four groups (three pens per group). Two groups were recorded each week, resulting in an alternating recording schedule. On week 0, a baseline RFID recording was taken, followed by the application of treatments (represented as scissors in the diagram). Recordings were then taken every two weeks post-treatment application for six weeks.

RFID technology (Biomark^®^, Boise, Idaho, USA) was used to determine the amount of time that individual hens spent at elevated resources. RFID recordings took place bi-weekly, over eight weeks, for 48 h intervals beginning with week 0 (baseline measurement before treatment application) for a total of four measurements per hen (week 0, 2, 4, 6; figure 3). Plastic leg bands (20 mm) fixed with individual passive integrated transponder (PIT) tags (Biomark^®^ HPT 12 PIT tag, 12.5 mm, 134.2 kHz) were assigned to each hen. Antennas (30 × 10 × 2 cm) (Biomark^®^, Boise, Idaho, USA) generating a radio-frequency field were fixed to feeders and placed within nest-boxes. PIT tags were activated by the radio-frequency field of individual antennas as hens accessed feeders and nest-boxes.

**Figure 4.**
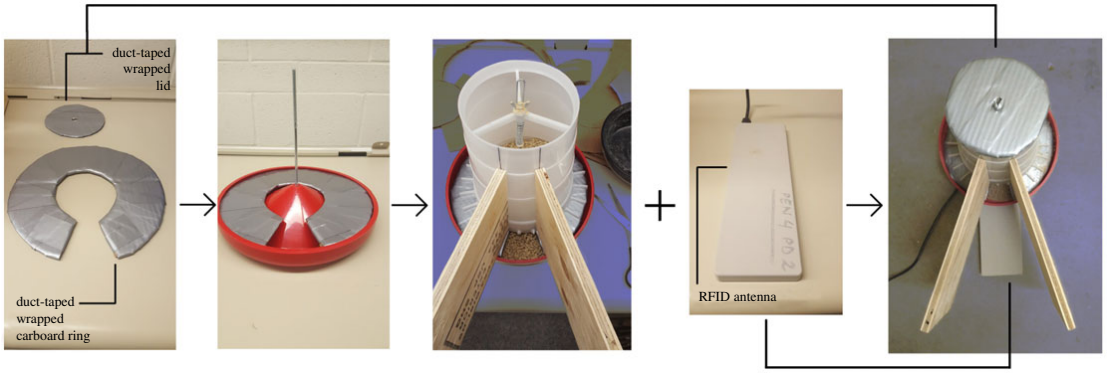
Individual feeder assembly. A duct-taped wrapped cardboard ring was placed into the base of a 5 kg hanging feeder. Two wooden boards were aligned with the opening in the ring and were secured onto the body of the feeder with four screws. An RFID antenna was attached to the base of the feeder with plastic zip ties, and the cardboard wrapped lid was placed atop the opening at the top of the feeder.

Hens had access to two 5 kg hanging feeders (Frandsen Corporation, North Branch, Minnesota, USA): one located on the ground and the other located at an elevated position at the front end of platform 1 (figure 1). Similarly, each pen contained two individual nest-boxes, one elevated at the front end of platform 2 and the second on the ground (figure 1). To prevent interference with RFID recordings, it was imperative that only one hen could access each feeder and nest-box at a time. Feeders were adapted to ensure individual access. Duct-tape (Gorilla Tape; The Gorilla Glue Company, Ohio, USA) wrapped cardboard rings were placed inside all feeders to alter the feed flow to one spot. Two wooden boards were placed on either side of this so that only one hen could step onto the antenna and access the feeder at one time (figure 4).

This resulted in recording a timestamp (HH:MM:SS:MS) for every millisecond a PIT tag was in the range of the antenna's field. Recordings were stored on a microchip and later transferred onto a computer. Raw files obtained from the RFID unit were run through a custom script (Perl V. 5.28.0) that output the total duration (milliseconds) spent at each feeder and nest-box for each hen. The Perl script used a threshold of 60 000 ms (1 min) to differentiate between two separate visits to the resource by the same hen. The script generated tabular data that included information on the individual hen ID, pen, date and total duration of the visits to the resource associated with the specific antenna.

### 2.4. Statistical analysis

The total duration spent at each resource (feeders or nest-boxes) was calculated by summing the amount of time spent at the ground and elevated resource. We calculated the percentage of time that a laying hen spent at the elevated resource out of the total time spent at the respective resource (i.e. percentage of time at elevated feeder and percentage of time spent at the elevated nest-box).

Data analysis was conducted using SAS Version 9.4 (SAS Institute Inc., Cary, NC, USA, 2012) using a generalized linear mixed model procedure (PROC GLIMMIX). The assumptions of normally distributed residuals and homogeneity of variance were assessed using studentized residual plots and the Shapiro–Wilk statistic to determine the statistical distribution that best fits the data. No deviation from normality was detected and no transformations of the data were performed.

Response variables used were (i) percentage of time spent at the elevated feeder and (ii) percentage of time spent at the elevated nest-box out of the total time spent at the respective resource. The model included flight feather clipping status (control, half clip and full clip), strain (white-feathered, brown-feathered), week (baseline, week 2, 4, 6 after experimentally induced wing feather damage) and their interactions as fixed effects. The hen within each pen was considered as the experimental subject, and the model was adjusted for bi-weekly repeated measures. Degrees of freedom were calculated using a Kenward-Roger approximation. A Tukey-Kramer adjustment was used to account for multiple comparisons. A *p*-value of less than 0.05 was considered statistically significant for all presented results. Herein we report means ± s.d.

## 3. Results

### 3.1. Use of elevated resources before flight feather clipping in white- and brown-feathered hens

Before treatments were applied (baseline), white-feathered hens spent significantly more (F_1,10.04_ = 7.35, *p* = 0.0218) time feeding at the elevated feeder than brown-feathered hens (53.4 ± 6.51% versus 24.0 ± 6.51%). By contrast, both white-feathered hens (72.14 ± 7.68%) and brown-feathered hens (68.00 ± 8.26%) spent an equal amount of time at the elevated nest-box before their flight feathers were clipped.

### 3.2. Use of elevated resources after flight feather clipping in white-and brown-feathered hens

Clipping status affected elevated feeder use in white-feathered hens (table 1). We detected a 41.8% decline in elevated feeder use two weeks after flight feathers were fully clipped (*t*_169.9_ = 5.97, *p* < 0.0001); however, no such decline was observed in brown-feathered hens.

**Table 1.** Percentage of time that white- and brown-feathered hens (*n* = 120) spent at the elevated feeder and elevated nest-box (calculated from the total number of minutes at the ground + elevated feeder, or nest-box) on week 0 week (baseline week before treatments were applied), and two, four and six weeks after treatment application. Within each treatment group, identical letters (a or b) represent least squared means that are statistically similar to one another.

strain	white-feathered			brown-feathered		
	control	half clip	full clip	control	half clip	full clip
weeks	(%)	(%)	(%)	(%)	(%)	(%)
elevated feeder						
0	46.39 ± 7.98%^a^	48.60 ± 8.80%^a^	65.31 ± 8.70%^a^	21.38 ± 7.76%^a^	29.04 ± 8.35%^a^	21.48 ± 8.20%^a^
2	48.84 ± 7.98%^a^	51.17 ± 8.70^a^	23.56 ± 8.70%^b^	28.44 ± 7.82%^a^	10.04 ± 8.48%^a^	10.51 ± 8.42%^a^
4	42.38 ± 7.98%^a^	46.41 ± 8.70%^a^	23.25 ± 8.70%^b^	24.06 ± 9.32%^a^	13.06 ± 10.10%^a^	9.37 ± 10.49%^a^
6	54.96 ± 7.98%^a^	38.62 ± 8.70%^a^	26.15 ± 8.70%^b^	37.21 ± 7.90%^a^	15.17 ± 8.61%^a^	18.35 ± 8.55%^a^
elevated nest-box						
0	63.50 ± 9.30%^a^	75.98 ± 10.25%^a^	76.95 ± 10.10%^a^	55.13 ± 10.58%^a^	77.46 ± 11.54%^a^	71.40 ± 11.48%^a^
2	63.89 ± 9.30%^a^	78.38 ± 10.10%^a^	39.70 ± 10.10%^b^	48.45 ± 10.58%^a^	23.19 ± 11.73%^b^	12.89 ± 11.48%^b^
4	62.96 ± 9.30%^a^	66.92 ± 10.10%^a^	26.33 ± 10.10%^b^	45.05 ± 12.60%^a^	31.71 ± 14.21%^b^	9.67 ± 14.78%^b^
6	65.24 ± 9.30%^a^	62.01 ± 10.10%^a^	16.24 ± 10.10%^b^	40.16 ± 10.68%^a^	17.51 ± 12.13%^b^	15.53 ± 12.06%^b^

The patterns seen on week 2 for the percentage of the time spent at the elevated feeder for both strains can also be seen four and six weeks after treatments were applied. Full clip white-feathered hens spent approximately 25% of their time feeding at the elevated feeder on week 4 (−42.1% from week 0, *t*_169.9_ = 6.01, *p* < 0.0001) and week 6 (−39.2% from week 0, *t*_169.9_ = 5.60, *p* < 0.0001). For the remaining white-feathered hens, the control group (40–55%) and the half clip group (40–50%) did not change the percentage of time spent at the elevated feeder compared to baseline (all *p* > 0.05). Similarly, the brown-feathered hens did not differ in the percentage of time spent at the elevated feeder from baseline regardless of treatment. However, it is interesting to note that the half clip and full clip groups showed numerical decreases, while birds in the control group showed numerical increases over the weeks spent at the elevated feeder compared to week 0.

The percentage of time spent at the elevated nest-box differed depending on the strain and clipping status two weeks after treatments were applied. For white-feathered hens, the control and half clip group did not differ in the percentage of time spent at the elevated nest-box. However, the full clip group spent 37% less time at the elevated nest-box compared to week 0 (*t*_170.2_ = 4.12, *p* = 0.0029). Both clipping treatments resulted in a reduction of time hens spent at the elevated nest-box in brown-feathered hens two weeks after treatments were applied. Both clipping treatments reduced the time at the elevated nest-box by more than 50%, with a stronger decrease in the full clip group (*t*_124.1_ = 5.96, *p* < 0.0001) than the half clip group (*t*_121.5_ = 5.48, *p* < 0.0001). The control group spent the same amount of time at the elevated nest-box as before treatments were applied at week 0.

The patterns of elevated nest-box usage that emerged as a result of treatment application on week 0 remained consistent four and six weeks after treatment application. For the white-feathered hens, the control group and half clip group did not differ from week 0 in the amount of time spent at the elevated nest-box (all *p* > 0.05). However, the full clip group spent less time at the elevated nest-box on week 4 (−50.6% from week 0, *t*_170.2_ = 5.60, *p* < 0.0001) and week 6 (−60.7% from week 0, *t*_170.2_ = 6.72, *p* < 0.0001). For the brown-feathered hens, the control group spent the same amount of time at the elevated nest-box over all weeks (all *p* > 0.05). However, both clipping treatment groups showed a major decrease in the amount of time spent at the elevated nest-box, with the half clip group nearly halving the amount of time on week 4 (−45.8% from week 0, *t*_126.3_ = 3.60, *p* = 0.0195) and more than halving the amount of time on week 6 (−60.0% from week 0, *t*_122.9_ = 5.78, *p* < 0.0001). Finally, the full clip group spent between 10–15% of their time at the elevated nest-box on week 4 (−61.7% from week 0, *t*_126.9_ = 4.60, *p* = 0.0004) and week 6 (−55.9% from week 0, *t*_123.1_ = 5.39, *p* < 0.0001).

## 4. Discussion

When all flight feathers were clipped, laying hens spent significantly less time accessing elevated resources (table 1). Additionally, brown-feathered laying hens were the most affected; showing a decline in elevated resource use when both primary and secondary flight feathers were clipped (full clip) and when only primary flight feathers were clipped (half clip). By contrast, white-feathered hens were only affected by the full clip treatment. Lastly, both strains showed a higher usage of the elevated nest-box from the start, which was reduced significantly after hens had their flight feathers clipped.

All hens who received the full clip treatment had a noticeable decline in time spent accessing elevated resources. Provine *et al*. [29] showed that as a species, domestic chickens possess wings that are not large enough to support their body weight, resulting in high wing loading and poor flight ability. When hens had fully clipped flight feathers, wing area was reduced by approximately 55% per wing. It is likely that by further reducing wing area, laying hens were not able to accommodate an increase in wing loading and were physically restricted in their ability to move upwards. Alternatively, a reduction in wing area would require an increase in flight muscle power for a bird to support its body weight [30]. The chicken pectoralis contains mostly white muscle fibres which fatigue rapidly [31]. It is possible that an increase in power output would result in more rapid fatigue of the flight muscles, and hens may opt to use ground resources to avoid associated energetic costs; however, this would require further investigation. In addition to an increase in wing loading, lost and damaged flight feathers are known to result in stability challenges [25], reduced aerodynamic efficiency [7] and poor maneuverability [19]. All these factors may cause a reduction in elevated resource access. While these consequences would make flying upwards to access resources difficult, they may also result in challenges in descending safely from platforms. Even when fully feathered, it has been shown that laying hens descend at a velocity of approximately 3.94 m s^−1^ [19], compared to the 0.92 m s^−1^ and 0.85 m s^−1^ of the Zebra Finch (*Taeniopygia guttata*) and Diamond Dove (*Geopelia cuneata*), respectively [32]. This means that laying hens move downwards at a fast rate, which can result in injuries or pain upon impact with the ground. This may be further exacerbated if laying hens feed on the elevated feeder and subsequently carry additional weight within their crop, increasing their mass and possibly increasing their descent velocity. Thus, hens may have decreased the amount of time spent at elevated resources to avoid discomfort associated with platform descent.

The increased use of ground resources suggests that laying hens cannot make adjustments to accommodate lost wing area. This differs from some species of flying birds that will physiologically or mechanically adjust to maintain flight capabilities when wing area is lost. For example, the ruby-throated hummingbird has been shown to reduce body weight by up to 25% to compensate for increased wing loading during flight feather moult [33]. And starlings with experimentally removed flight feathers will shift from an elliptical to a figure-eight wingtip trajectory to accommodate for lost wing areas [34]. Many species of flying insects are subject to loss of wing area through collisions [35,36] and predatory attacks [37]. While this may result in decreased manoeuvrability, insects are able to adjust flapping kinematics such as flapping frequency [38–40], flapping amplitude [39,40] and stroke plane angle [41] to maintain flight ability. As the cuticle of insect wings appears to lack healing ability [42], it seems reasonable that evolutionary adaptations to accommodate wing area reduction during flight are under strong selection. This would not be important for the survival and fitness of commercial laying hens and highlights a possible reason for hens being poor flyers.

Ground-level feeders were used over elevated feeders in brown-feathered birds regardless of clipping treatment. This result expands on the general results of other studies where brown-feathered birds are less aerial than white-feathered birds [14,16,43]. Unexpectedly, there was no difference in half-clipped and fully clipped brown-feathered birds in their usage of the ground feeder. We expected that the increase in flight feather clipping (from 33% to 55%) would increase ground-level feeder use; however, it might be that a more than 20% wing area loss was not linearly proportional to the reduction in elevated feeder usage. On the other hand, the clipping treatment applied in the present experiment may not have been sufficient to change behaviour. Additionally, it is possible that brown-feathered hens who received a clipping treatment may have had a strong ground-level feeder preference prior to the clipping treatment. White-feathered fully clipped hens were the only group to spend significantly less time at the elevated feeder and nest-box after treatments were applied. The half-clipped and control white-feathered hens maintained the same pattern of use before and after treatment application. The white-and brown-feathered hens used in this trial came from different rearing environments which may have affected baseline preferences for ground-level and elevated resources.

The full clip (white- and brown-feathered hens) and half clip (brown-feathered hens) treatments significantly reduced the amount of time that laying hens spent at the elevated nest-box. Hens are highly motivated to lay an egg within a secluded nest-box [44]. In multi-tiered housing systems, nest-boxes are typically seen on the upper tiers. Our results indicate that when hens were missing flight feathers, they transitioned to primarily using nest-boxes on the ground. This suggests that hens with clipped flight feathers had a reduction in flapping flight performance. Not only may this result in more eggs laid on the ground, an undesirable outcome for farmers, but it may also be a source of frustration for laying hens. Additionally, in the case that many hens within a flock have missing or damaged flight feathers, it may be that a larger proportion of hens will remain on the ground. This could result in overcrowding and the inability of hens to make use of the full three-dimensional space in the rearing system.

In using RFID technology, we were able to record the amount of time that a hen spent within the range of specific antennas placed at specific locations, as well as the total number of visits to these locations. This technology is, however, not without limitations as it does not allow us to know how much time birds spent on platforms and perches away from antenna sites, which behaviours were performed at antenna sites, or how birds locomoted to different areas (jumping, flapping, etc.). For these same reasons, the data acquired for the number of visits to each antenna location was not used as we could not differentiate a hen who ascended to a platform one time and made multiple visits to an elevated resource from a hen who ascended multiple times to access a resource the same amount of times. Additionally, as there were no other behavioural measurements, we cannot completely rule out that when clipped birds spent more time at the ground resources, it resulted in an increase in competition for these resources, forcing non-clipped birds to move up to use elevated resources. However, our data suggest that this is not the case as non-clipped birds showed the same pattern of feeder usage throughout the trial. Similarly, we cannot rule out the possibility of the feather clipping treatments potentially disrupting the social hierarchy of the group of birds within each pen and altering resource usage. Combining RFID data with behavioural observations would be an interesting approach to elucidate some of these effects. However, as hens had been living together in the experimental set-up four months prior to the beginning of the trial and were already aged 34 and 39 weeks of age, it is likely that the social hierarchy was already well established. Further, personal observations of the birds throughout the duration of the trial did not indicate increased levels of aggression or competition between the differently clipped birds.

We used symmetric flight feather clipping to simulate wing feather loss/damage. However, not all feather loss is equal, and we assume symmetric damage is less likely to occur naturally. To improve understanding of the effects of asymmetrical feather loss and loss of wing area due to wear, it would be useful for future research to investigate the effects of natural (i.e. unmanipulated) feather damage on resource use of hens who have incurred wing feather loss and damage within their housing environment. In general, studies of flight in birds focus primarily on the wings [32,45,46] while the tail is largely underrepresented. However, tail feathers are a heavily targeted area of feather pecking behaviour [47–49] that may significantly affect upwards mobility of laying hens. The tail feathers play a role in generating force during takeoff, stability, balance and braking during landing [50,51]. As such, next steps should include investigations of tail feather loss on flight kinematics and resource usage of laying hens.

## 5. Conclusion

We found significant evidence of change in resource usage of laying hens with clipped flight feathers. We showed that when all flight feathers were clipped, laying hens reduced the use of elevated feeders and nest-boxes in favour of ground resources. Additionally, heavier, brown-feathered birds appeared more affected by experimentally induced feather loss than white-feathered birds. Our results can have an impact on density, accessibility and spatial distribution of resources in backyard coops and farms and point to changing the layout of housing systems in favour of hens' abilities.

## Ethical

This study was approved by the University of Guelph Animal Care Committee (Animal Utilization Protocol # 3908) prior to the start of data collection.

## Data Availability

Raw data have been made publicly available. The data are provided in the electronic supplementary material [52].
